# Racial differences in blood pressure variability in the SPRINT trial

**DOI:** 10.1002/alz.086458

**Published:** 2025-01-09

**Authors:** Isabel Sible, Daniel A. Nation

**Affiliations:** ^1^ University of Southern California, Los Angeles, CA USA; ^2^ Keck School of Medicine, Los Angeles, CA USA

## Abstract

**Background:**

Blood pressure (BP) management is an accessible therapeutic target for dementia prevention. BP variability (BPV) is a newer aspect of BP control recently associated with cognitive decline, dementia and Alzheimer’s disease (AD), independent of traditionally targeted mean BP levels. Most of this work has relied on largely non‐Hispanic White study samples in observational cohorts. It remains unclear what role BPV may play in dementia‐related racial/ethnic health disparities. We examined whether BPV differed by race in the SPRINT BP clinical trial.

**Method:**

In this post hoc analysis of the SPRINT trial, 8034 participants (median (SD) 67 (9.3) years; 34.8% female; 28.6% Black; 58.9% White; 10.8% Hispanic; 1.7% Other) underwent 4 BP measurements over a 9‐month period after treatment randomization (standard [<140 mmHg systolic target] vs intensive [<120 mmHg systolic target] BP lowering). Visit‐to‐visit systolic BPV was calculated as variability independent of mean. Racial groups (Black vs White vs Hispanic vs Other) were compared on BPV using ANCOVA models adjusted for age, sex, mean BP over the same 9‐month period, and number of antihypertensive medications used. Analyses were stratified by standard vs intensive treatment group.

**Result:**

Under standard BP lowering treatment, systolic BPV significantly differed by race (*F*_3,3969_ = 10.11, *p* < .001). When compared to Hispanic participants (mean (SD) 8.96 (1.1) mmHg), systolic BPV was higher in Black (9.26 (1.0) mmHg; .30 mmHg difference; *p* < .001), White (9.20 (1.0) mmHg; .24 mmHg difference; *p* < .001), and Other (9.31 (.9) mmHg; .35 mmHg difference; *p* = .01) participants. Racial groups did not significantly differ on systolic BPV under intensive BP treatment (*F*_3,3978_ = 1.41, *p* = .24).

**Conclusion:**

Although the SPRINT trial found no racial differences in treatment effects on cardiovascular benefits/risks, our findings suggest BPV differs by race, particularly under standard BP treatment. BPV is an emerging risk factor for dementia that may require unique treatment approaches beyond traditional lowering strategies. Findings could have therapeutic implications for patient‐centered care strategies aimed at reducing dementia risk and enhance our broader understanding of health disparities in vascular health relevant to AD risk.

